# A202 A CASE OF MYCOBACTERIUM WOLINSKYI PULMONARY INFECTION SECONDARY TO LONG-STANDING ACHALASIA IN AN IMMUNOCOMPETENT PATIENT

**DOI:** 10.1093/jcag/gwad061.202

**Published:** 2024-02-14

**Authors:** J C Bowron, D C Sadowski

**Affiliations:** Medicine, University of Alberta Faculty of Medicine & Dentistry, Edmonton, AB, Canada; Medicine, University of Alberta Faculty of Medicine & Dentistry, Edmonton, AB, Canada

## Abstract

**Background:**

Achalasia is an esophageal motility disorder resulting in chronic esophageal obstruction and stasis. Rapidly growing mycobacterium (RGM) pulmonary infections have been previously observed in individuals with underlying gastro-esophageal motility disorders. To date, pulmonary *Mycobacterium wolinskyi* infection *(MWI)* in the setting of achalasia has not previously been reported.

**Aims:**

Our aim is to present a novel case of *M. wolinskyi* pneumonia in a patient with achalasia. We also searched the medical literature to identify previously reported associations between achalasia, chronic regurgitation symptoms and MWI. *M. wolinskyi* is an emerging clinical concern among RGMs. While primarily associated with post-operative, prosthetic joint and skin and soft tissue infections, *M. wolinskyi* pulmonary infections have not been documented to date in an immunocompetent patient.

**Methods:**

We present a retrospective clinical case description of our case. We carried out a literature review from 1999 to Oct 2023, with the keywords "*M. wolinskyi* and infection.

**Results:**

A 34-year-old male presented with a several-month history of productive cough, exertional dyspnea, fatigue, and unintentional weight loss of 17 kg over the past 6 months. Despite multiple courses of oral and intravenous antibiotics, there was no clinical improvement. His medical history included long-standing achalasia requiring previous pneumatic dilations. On admission, his chest X-ray revealed bilateral interstitial and alveolar infiltrates with consolidation. Routine microbiology and viral panels were negative. CT scan showed widespread bilateral consolidation and a dilated and fluid-filled esophagus. A timed barium swallow demonstrated dilated esophagus with irregular tertiary contractions and limited GE-J opening.

Sputum cultures were positive for MWI on day 8 of admission (BACTEC MGIT). This was confirmed with lung biopsy demonstrating MWI with necrotizing granulomatous inflammation. The patient was prescribed a 12-month course of doxycycline, trimethoprim-sulfamethoxazole, and ciprofloxacin. Inpatient pneumatic dilation to 35 mm was performed. At discharge, his swallowing was subjectively improved, and a follow-up swallowing study noted improvement with no evidence of regurgitation.

**Conclusions:**

Chronic esophageal obstruction provides an ideal lipid-rich environment for RGM infections. Related mycobacterial species have been isolated in pulmonary infections have been linked to chronic esophageal obstruction. To date, no cases of *M. wolinskyi* pulmonary infection with achalasia have been reported. We present a unique case of *M. wolinskyi* pulmonary infection in an immunocompetent patient with chronic achalasia, adding to our understanding of this emerging clinical concern.

**Funding Agencies:**

None

